# Long Covid-19: Proposed Primary Care Clinical Guidelines for Diagnosis and Disease Management

**DOI:** 10.3390/ijerph18084350

**Published:** 2021-04-20

**Authors:** Antoni Sisó-Almirall, Pilar Brito-Zerón, Laura Conangla Ferrín, Belchin Kostov, Anna Moragas Moreno, Jordi Mestres, Jaume Sellarès, Gisela Galindo, Ramon Morera, Josep Basora, Antoni Trilla, Manuel Ramos-Casals, on behalf of the CAMFiC long COVID-19 Study Group

**Affiliations:** 1 Permanent Board of the Catalan Society of Family and Community Medicine (CAMFiC), 08009 Barcelona, Spain; lconangla.mn.ics@gencat.cat (L.C.F.); jordimestres@camfic.org (J.M.); 2 Primary Care Centre Les Corts, Consorci d’Atenció Primària de Salut Barcelona Esquerra (CAPSBE), 08028 Barcelona, Spain; badriyan@clinic.cat; 3 Primary Healthcare Transversal Research Group, Institut d’Investigacions Biomèdiques August Pi i Sunyer (IDIBAPS), 08036 Barcelona, Spain; 4 Laboratory of Autoimmune Diseases Josep Font, IDIBAPS-CELLEX, 08036 Barcelona, Spain; mbritozeron@gmail.com (P.B.-Z.); mramos@clinic.cat (M.R.-C.); 5 Autoimmune Diseases Unit, Department of Medicine, Hospital CIMA-Sanitas, 08034 Barcelona, Spain; 6 Department of Autoimmune Diseases, ICMiD, Hospital Clínic, 08036 Barcelona, Spain; 7 Department of Statistics and Operations Research, Universitat Politècnica de Catalunya (UPC), 08034 Barcelona, Spain; 8 Jaume I Health Centre, Institut Català de la Salut, Universitat Rovira i Virgili, 43005 Tarragona, Spain; Anamaria.moragas@urv.cat; 9 College of Catalan Physicians, 08017 Barcelona, Spain; jaume.sellares@comb.cat; 10 Permanent Board of the Spanish Society of Family and Community Medicine (semFYC), 08009 Barcelona, Spain; ggalindoo@semfyc.es; 11 Board of Spanish Society of Managers of Primary Care (SEDAP), 28026 Madrid, Spain; dr.rmorera@gmail.com; 12 IDIAP Jordi Gol, 08007 Barcelona, Spain; jbasora@idiapjgol.org; 13 Faculty of Medicine and Health Sciences, University of Barcelona, 08036 Barcelona, Spain; ATRILLA@clinic.cat

**Keywords:** SARS-CoV-2, primary care, long COVID-19

## Abstract

Long COVID-19 may be defined as patients who, four weeks after the diagnosis of SARS-Cov-2 infection, continue to have signs and symptoms not explainable by other causes. The estimated frequency is around 10% and signs and symptoms may last for months. The main long-term manifestations observed in other coronaviruses (Severe Acute Respiratory Syndrome (SARS), Middle East respiratory syndrome (MERS)) are very similar to and have clear clinical parallels with SARS-CoV-2: mainly respiratory, musculoskeletal, and neuropsychiatric. The growing number of patients worldwide will have an impact on health systems. Therefore, the main objective of these clinical practice guidelines is to identify patients with signs and symptoms of long COVID-19 in primary care through a protocolized diagnostic process that studies possible etiologies and establishes an accurate differential diagnosis. The guidelines have been developed pragmatically by compiling the few studies published so far on long COVID-19, editorials and expert opinions, press releases, and the authors’ clinical experience. Patients with long COVID-19 should be managed using structured primary care visits based on the time from diagnosis of SARS-CoV-2 infection. Based on the current limited evidence, disease management of long COVID-19 signs and symptoms will require a holistic, longitudinal follow up in primary care, multidisciplinary rehabilitation services, and the empowerment of affected patient groups.

## 1. Introduction

Severe acute respiratory syndrome 2 coronavirus (SARS-CoV-2), first detected in December 2019 in Wuhan, China, is the seventh coronavirus known to infect humans after the identification, in this century, of SARS and Middle East Respiratory Syndrome (MERS) viruses. The lack of pre-virus immunity has led to an exponential increase in infected patients worldwide and the pandemic is one of the biggest health challenges facing humanity in the last 100 years [1,2]. The rapid and unpredictable worldwide spread of SARS-CoV-2, with most infected people having no or only mild signs and symptoms, appears to have initially been related to cases imported from the first countries affected [3]. As of January 13, 2021, there are >90 million confirmed cases worldwide and 2 million deaths [4].

COVID-19, the disease caused by SARS-CoV-2, has a very broad clinical spectrum. The most common clinical presentation of COVID-19 is mild respiratory infection and, less commonly, pneumonia with fever, cough and dyspnea [1,2]. Other signs and symptoms, such as odynophagia, anosmia, ageusia, muscle aches, diarrhea, chest pain and headaches, among others, are part of the acute infection (Table 1) [5,6,7,8,9,10,11,12,13,14,15,16,17,18,19]. The main studies show a mean symptom duration ranging from 11 days [12,20] to 28 days in patients admitted to the ICU [21]. According to a recently published study by Rubio-Rivas et al. [22] the ICU admission rate was 9.3% in the general population. The abnormal persistence of signs and symptoms for >4 weeks after the resolution of SARS-CoV-2 infection has been little studied, and there are no studies in primary care, where most COVID-19 diagnoses are made.

The objective of this document was to develop primary care clinical guidelines for patients with long COVID-19 signs and symptoms to enable primary care professionals to address the health consequences that go beyond acute SARS-CoV-2 infection.

## 2. Definitions

In the absence of consensus and internationally accepted definitions, we used the recently-proposed definitions in the National Institute for Health and Care Excellence (NICE) working document, based on the effects of COVID-19 at different time points [23]:

- Acute COVID-19 infection: Signs and symptoms of COVID-19 for up to 4 weeks. We support the definition of COVID-19 infection included in the NICE guidelines [23], including people with suspected infection who, in the early phases of the pandemic, did not have access to testing for SARS-CoV-2 infection.
- Ongoing symptomatic COVID-19: Signs and symptoms of COVID-19 from 4 to 12 weeks not explained by an alternative diagnosis after protocolized study.
- Post-COVID-19 syndrome: Signs and symptoms that develop during or following an infection consistent with COVID-19, continue for >12 weeks and are not explained by an alternative diagnosis. The term “syndrome” reflects the concurrence of a multisystem, fluctuating, and often overlapping clusters of signs and symptoms that, in some patients, may follow a relapsing-remitting pattern and that may change over time and affect any bodily system.

## 3. Methodology

In the absence of evidence-based clinical practice guidelines (CPGs) for the management of long COVID-19 [24,25], Catalan Society of Family and Community Medicine (CAMFiC) established a working group to develop a CPG, consisting mainly of primary care professionals (90%), together with specialists in internal medicine, autoimmune diseases, infectious disease, epidemiology and statistics.

The CPG focuses on patients with long COVID-19 not requiring hospitalization, whose diagnosis and follow-up has been made in primary care (probably > 80% of affected people). It does not focus on hospitalized patients, whose follow-up and management will be carried out by the hospital outpatient department.

In these guidelines, we also used the term “Long COVID-19”, which includes both ongoing and post-COVID-19 syndrome according to the NICE definitions [23]. We endorse the rationale for the terms used by the NICE guidelines, whose intention is to reflect the fact that signs and symptoms occur after the acute infection is ended (but not that the person has recovered), avoiding time-specific terms like “chronic” or “persistent”.

Signs and symptoms present during the first 4 weeks of infection, worsening or recurrence of manifestations already present before infection, manifestations arising from physical or psychic sequelae reasonably attributable to infection, and post-viral immune-related manifestations not initially present and which may appear once the infection is cured fall outside the scope of the guidelines [26].

We used a pragmatic approach based on the few published studies on SARS-CoV-2, editorials and expert opinions, press releases and the clinical experience of the authors. Academic sources were identified by a systematic search of the PubMed database, through 13 January 2021, with the main terms SARS-CoV-2 and COVID-19 in combination with the secondary terms chronic, persistent, ongoing, long-term, recovery time, and post-viral, along with specific searches for each individual symptom included in the guidelines.

## 4. Planning of Care for Patients with Long Covid-19

The care of patients with long COVID-19 should be structured in three consecutive visits according to the time from diagnosis of SARS-CoV-2 infection.

The first primary care visit (V1) of patients with long COVID-19 is essential. The objective should be a history and examination and complementary tests to study the possible underlying causes of long symptoms. We endorse the recommendations suggested by the NICE guidelines for assessing people with long COVID-19 [23]. The visit should be made from the 4th week after confirmation of the diagnosis of SARS-CoV-2 infection with a positive SARS-CoV-2 test (PCR, antigen or antibody) or after the start of signs and symptoms of COVID-19 in case laboratory test is unavailable (preferably between the 5th and 6th week, depending on availability and resources), and should last at least 30 min, with active support from nurses, including:

Personal background: The medical record may be relevant when analyzing long-term symptoms. The family physician has the most comprehensive long-term information on the pre-infection health status.

SARS-CoV-2 infection: Diagnostic confirmation of SARS-CoV-2 infection (date and microbiological test), symptoms and approximate onset dates, hospital admission and discharge dates, maximum oxygen requirements, ICU admission and duration, therapies received, and complications during admission should be recorded. The intensity of each symptom may be assessed subjectively on a visual analog scale (VAS: 0–10). The diagnostic algorithms proposed for each individual symptom in paragraph IV will apply.

Physical examination: A complete physical examination, with measurement of vital signs and baseline oxygen saturation, should be made, paying special attention to assessing the oropharynx and cardiorespiratory system.

Laboratory studies: A basic first visit laboratory study should be made, supplemented according to individualized patient criteria (Table 2).

Complementary tests: Lung parenchyma evaluation is essential in all patients with COVID-19. Chest X-ray in at least the two conventional projections is the conventional test, and allows for agile general evaluation, and is usually accessible urgently. However, in primary care, chest ultrasound should be used when possible, as it is very useful in evaluating pneumonia and its complications (as shown in hospitals) [27] and in the differential diagnosis. It is accessible, may be used in outpatients, at home or in nursing homes, and is useful for the diagnosis and subsequent monitoring. Chest ultrasound may show peripheral pulmonary involvement, pulmonary interstitial disease (focal or diffuse), pleural contact condensations (pneumonic or thromboembolic), pneumothorax and pleural effusion. Other complementary tests will be determined by the symptoms presented (Table 2).

The second visit (V2) should be made from week 8 (preferably between weeks 9 and 10, depending on availability and resources). The objective is to evaluate the results of the V1 tests, make a differential diagnosis with other post-COVID-19 situations, and apply the corresponding diagnostic algorithms to identify potential causes that reasonably explain the symptoms.

The third visit (V3) should be made from week 12 (between week 13 and week 14 week, depending on availability and resources) to evaluate the evolution of long-term symptoms and re-evaluate possible causes using the corresponding diagnostic algorithms.

## 5. Assessment of Individual Signs and Symptoms

Primary care evaluations should follow the same principles as normal clinical practice for the same symptoms: a careful history that considers the medical record and a physical examination centered on the reported symptoms.

### 5.1. Fatigue

Fatigue is one of the most common extra-respiratory symptoms of acute SARS-CoV-2 infection (41%, Table 1). Studies estimate the frequency as 35–45% at 4 weeks [28,29], 30–77% at 8 weeks [30,31,32] and 16–55% at 12 weeks post-infection [33,34] (Table 3). The profound, prolonged nature of fatigue in some COVID-19 patients shares characteristics with the chronic fatigue syndrome (CFS) described after other infections, including SARS, MERS and community-acquired pneumonia [24,25]. No association has been reported between the long-term fatigue associated with COVID-19 and other long-term fatigue states, or with COVID-19 severity, or with any laboratory marker of inflammation and cell turnover or pro-inflammatory molecules. However, women and people with a pre-existing diagnosis of depression/anxiety were over-represented in patients with long-term fatigue [35].

Algorithm S1 summarizes the diagnostic approach for patients with fatigue persisting >4 weeks after SARS-CoV-2 infection. In these patients, V1 should include (File S1):

Specific clinical history: The history should record the onset date and specific questions about fatigue (symptoms and accompanying signs, concomitant psychosocial and emotional factors, related drugs, substance abuse, sleep disorders and exposure to toxins), pre-COVID-19 infection diseases possibly associated with chronic fatigue, organ-specific sequelae resulting from severe COVID-19 infection requiring hospital admission that may cause fatigue, and other current symptoms coexisting with fatigue.

Specific studies: Tests should include specific laboratory tests (chloride, bicarbonate, calcium, phosphate, muscle enzymes, plasma cortisol levels) and spirometry under safe conditions (according to the recommendations given by The Italian Respiratory Society for lung function testing in the context of COVID-19 [36]).

### 5.2. Arthralgia

Patients with acute SARS-CoV-2 infection may develop arthralgia (7.5%) (Table 1), defined as non-arthritic pain in ≥1 joints without evidence of inflammation (edema, joint pain or heat). It may be accompanied by difficult-to-locate muscle pain (arthromyalgia, musculoskeletal pain). Joint pain may persist in 10–15% of patients at 4 weeks [28,37] and 16–27% at 8 weeks [31,37] (Table 3).

Algorithm S2 summarizes the diagnostic approach in patients with joint pain persisting >4 weeks after SARS-CoV-2 infection (File S1). In these patients, V1 should include:

Specific clinical history: Record date of onset of joint pain, type of pain (nociceptive, neuropathic or mixed), location, duration, modification with exercise or rest (factors that relieve, worsen or trigger it) and response to analgesia. Pre-COVID-19 diseases possibly associated with joint pain and current symptoms co-existing with arthralgia (especially chronic fatigue) should be evaluated.

Specific studies: Tests should include specific laboratory tests (uric acid, proteinogram, antinuclear antibodies [ANA], rheumatoid factor, complement C3 and C4 levels). If joint inflammation is suspected, joint ultrasound is indicated (or simple radiology if not available). Monitoring of inflammation (synovitis and enthesitis) and peripheral joint damage may be useful, although there are insufficient data to recommend a specific ultrasound evaluation system or its periodicity.

### 5.3. Myalgia

Muscle pain or myalgia may affect ≥1 muscles and is usually benign and self-limiting. Ligaments, tendons, and fasciae may also be involved. In large series, myalgia is reported in 20% of cases of acute SARS-CoV-2 infection (Table 1) and may persist in 15% of patients at 4 weeks [29], in 6–13% at 8 weeks [30,31] and in 16% at 12 weeks [34] (Table 3).

Algorithm S3 summarizes the diagnostic approach to myalgia persisting >4 weeks after SARS-CoV-2 infection (File S1). In these patients, V1 should include:

Specific clinical history: Record the onset date, location, duration, modification with exercise or rest, factors that relieve, worsen or trigger myalgia, response to analgesia, pre-COVID-19 infections possibly associated with myalgia, and other current symptoms co-existing with myalgia (especially chronic fatigue and generalized pain).

Specific studies: The following specific laboratory tests should be added (proteinogram, creatine kinase, aldolase, lactate dehydrogenase, ANA, rheumatoid factor).

### 5.4. Chest Pain

Chest pain, defined as any pain located between the diaphragm and the base of the neck, appears in up to 13% of acute SARS-CoV-2 infections (Table 1). Chest pain may persist in 20% of patients at 4 weeks [28], in 22% at 8 weeks [31] and in 11% at 12 weeks [34] (Table 3). No studies have specifically described the characteristics of long-term chest pain in COVID-19. In our clinical experience, a significant percentage of patients refer to high central chest pain, a symptom described in a large patient-led survey as “pulmonary burning,” a kind of burning sensation in the chest, tension and some shortness of breath, especially reported after a dry cough [38].

Algorithm S4 summarizes the diagnostic approach to chest pain persisting >4 weeks after SARS-CoV-2 infection (File S1). In these patients, V1 should include:

Specific clinical history: Collect the date of onset, location, duration, triggers, modification with exercise or rest, accompanying symptoms, history of trauma or fall.

Specific studies: The following tests should be added: laboratory tests (troponins and creatine phosphokinase (CPK) -MB depending on availability), electrocardiogram, chest ultrasound (shown to be useful in the differential diagnosis of pleuritic pain to distinguish whether its origin is in the chest wall or lung surface) [39], and spirometry (under safe conditions); chest computed tomography (CT) evaluation may be considered.

### 5.5. Cough

Cough occurs in about 80% of acute SARS-CoV-2 infections (Table 1), while long-term cough has been reported in 33–43% of cases at 4 weeks [28,29], in 5–46% at 8 weeks [30,31,32], and in 2–17% at 12 weeks [33,34] (Table 3).

The diagnostic approach to patients with cough persisting >4 weeks after SARS-CoV-2 infection is summarized in Algorithm S5 (File S1). In these patients, V1 should include:

Specific clinical history: Collect the date of onset and characteristics (mostly dry, irritating, nonproductive cough; in productive cough the sputum characteristics should be investigated). Possible organ-specific sequelae resulting from severe COVID-19 infection requiring hospital admission that may cause chronic coughing, and iatrogenic sequelae related to invasive maneuvers (post-intubation orotracheal, post-tracheostomy) should be evaluated. Current symptoms co-existing with cough, especially new-onset fever and dyspnea, and other alarming symptoms should be evaluated.

Specific studies: Spirometry (under safe conditions) is advised.

### 5.6. Dyspnea

Dyspnea, the feeling of shortness of breath and difficulty in breathing properly, is sometimes confused with fatigue, and may be difficult to describe, for sociocultural reasons. Dyspnea is reported in 43% of patients with acute SARS-CoV-2 infection (Table 1). Long-term dyspnea is reported in 11–33% of cases at 4 weeks [28,29,37], in 8–63% of cases at 8 weeks [30,31,32,37], and 14% beyond 12 weeks [33] (Table 3).

Algorithm S6 summarizes the diagnostic approach to patients with dyspnea persisting >4 weeks after SARS-CoV-2 infection (File S1). In these patients, V1 should include:

Specific clinical history: Collect the data of onset and characteristics. It is important to rule out acute onset and to evaluate an association with increased physical demand or dyspnea at rest and, especially, an association with other symptoms, such as chest pain. The modified Medical Research Council (MRC) dyspnea scale may be administered. Organ-specific sequelae of severe COVID-19 infection requiring hospitalization that may cause dyspnea, invasive maneuvers and techniques carried out during the acute episode and which may have been an iatrogenic cause of secondary dyspnea, should be ruled out. Current symptoms co-existing with dyspnea, especially new-onset fever, should be assessed.

Specific studies: Additional laboratory tests should be added (troponins and CPK-MB, natriuretic peptides according to availability). Gasometry is recommended if baseline oxygen saturation is persistently decreased without known prior cause, respiratory functional tests (simple spirometry and diffusing capacity of carbon monoxide [DLCO]), chest radiology, and six-minute walk test (6MWT). CT or angio-CT should be considered.

Sudden-onset dyspnea (or baseline dyspnea flare-up) usually requires urgent attention, especially if associated with alarming symptoms, paying particular attention to respiratory superinfections, pulmonary thromboembolism (especially in patients with a history of hospitalization and severity), post-COVID-19 heart failure and organizing pneumonia. Late development of new respiratory symptoms and opacities (>2 weeks after the first symptoms of COVID-19), especially if not detected in previous CT studies, may suggest post-viral organizing pneumonia (already described in patients with influenza virus infection).

### 5.7. Anosmia/Dysgeusia

Partial (hyposmia) or complete (anosmia) loss of smell may be temporary or permanent, depending on the cause. Almost all patients with anosmia perceive salty, sweet, acid, and bitter substances normally, but do not discriminate flavors, which also depend heavily on smell. Therefore, patients refer to loss of the sense of taste (dysgeusia/ageusia) and do not enjoy food. Viral upper respiratory tract infection is a common cause of olfactory dysfunction, in part because the olfactory epithelium is adjacent to the respiratory epithelium, where many viruses that cause upper respiratory infection replicate, and partly because olfactory neurons directly access the environment. These viruses could cause olfactory dysfunction not only through nasal obstruction, but also through transient or persistent direct damage to the sensory epithelium. Anosmia after viral infection is known as post-infectious or post-viral olfactory loss. Anosmia and dysgeusia are present in 8–9% of patients with acute SARS-CoV-2 infection (Table 1). Studies show a frequency of 12–56% of long-term anosmia at 4 weeks [28,29,40,41,42,43], 2–25% at 8 weeks [30,31,42] and 13–46% at 12 weeks [34,44], while for dysgeusia, the rates are 9–50% [28,29,40,43], 1–10% [30,31], and 11–31% [34,44], respectively (Table 3).

The diagnostic approach to patients with anosmia/dysgeusia >4 weeks after SARS-CoV-2 infection is summarized in Algorithm S7 (File S1). In these patients, V1 should include:

Specific clinical history: Collect the date of onset and characteristics and rule out previous disease (especially ENT and neurological).

Specific studies: A specific physical examination including a complete otolaryngological examination should be made.

### 5.8. Headache

Headache has been reported in 14% of SARS-CoV-2 infection patients (Table 1). Reports show a frequency of long-term headache of 14% at 4 weeks [28], 9–15% at 8 weeks [29,31] and 18% at 12 weeks [33] (Table 3).

Algorithm S8 summarizes the diagnostic approach to patients with headache persisting beyond 4 weeks after SARS-CoV-2 infection (File S1). In these patients, V1 should include:

Specific clinical history: Collect the date of onset and the main features. Evaluate manifestations leading to the suspicion of an underlying organic disease. A prior diagnosis of headache (reported in 50% of patients with long-term headache) [45] or neurological disease, and current symptoms coexisting with headache, especially neurological symptoms, should be evaluated.

Specific studies: The examination should include blood pressure, temporal artery inspection and palpation in patients aged >50 years, temporomandibular joint examination, cranial palpation (painful spots, paranasal sinus, examination of sensitive points and triggers) and a complete neurological assessment (level of consciousness and meningogenic signs, gait, dysmetria, Romberg test, facial asymmetry, funduscopy).

### 5.9. Digestive Signs and Symptoms

The overall rate of patients with acute SARS-CoV-2 infection with gastrointestinal symptoms is 34%, including anorexia (21%), diarrhea (13%), nausea or vomiting (12%) and abdominal pain (11%) [46] (Table 1). Diarrhea, the most common gastrointestinal clinical sign, usually consists of non-severe, non-dehydrating semi-liquid stools [46]. It is unclear what percentage of these patients received treatments that produced gastrointestinal side effects during the first pandemic wave. Reports show the persistence of diarrhea as 3–9% [29,31], anorexia as 8% [31], nausea as 6% [29] and abdominal pain as 3% at 8 weeks post-infection [29] (Table 3).

Algorithm S9 summarizes the diagnostic approach to patients with digestive symptoms persisting beyond 4 weeks after SARS-CoV-2 infection (File S1). In these patients, V1 should include:

Specific clinical history: Collect the date of onset and main features, previous gastrointestinal disease and former and current treatments.

Specific studies: The following tests should be added: laboratory tests (pancreatic enzymes, anti-transglutaminase tissue immunoglobulin A), determination of occult blood in feces, abdominal ultrasound, and assess digestive endoscopy, functional studies, and food intolerance.

### 5.10. Other Long-Term Signs and Symptoms

A wide range of long-term symptoms is reported (Table 3), including general symptoms (fever, chills, intolerance to temperature changes), otolaryngologic symptoms (rhinitis, nasal congestion, tinnitus, vertigo, pain, oropharyngeal discomfort), neuropsychological symptoms (confusion or “mental fog”, concentration and sleep disorders, instability), dryness or conjunctivitis. As with other stressful life situations or major illnesses, COVID-19 can lead to temporary intense hair loss (telogen effluvium) weeks after acute disease. There is not sufficient information to propose specific approaches to most of these symptoms, which are heterogeneous, unaccompanied by alterations in complementary tests, and generally too nonspecific to be attributed to a specific organic involvement. If there are normal results in the appropriate diagnostic tests, most could be included in syndromic presentations related to chronic upper airway involvement (as with other respiratory viral infections) or central sensitivity syndromes (such as CFS, FM, and SHQM).

## 6. Diagnostic Approach to Long Covid-19

The main objective of this CPG is to guide the clinical approach to patients with long COVID-19 in primary care following a protocolized study of the symptoms. Before establishing a probable diagnosis of post-COVID-19 syndrome, a differential diagnosis with other post-COVID-19 situations should be made once the corresponding diagnostic algorithms are applied to identify potential causes that may reasonably explain the symptoms.

### 6.1. Differential Diagnosis

The diagnostic approach to long COVID-19 should start by ruling out processes unrelated to SARS-CoV-2 infection. The standard primary care assessment should include the diagnosis of other, matching pathologies unrelated to the viral infection. In addition, primary care review of the medical record may identify pre-existing pathologies or symptoms which could be exacerbated after infection, always including the appropriate complementary tests to rule out other etiologies.

Other processes directly related to SARS-CoV-2 infection should be investigated as a potential origin of the long-term symptoms.

#### 6.1.1. Cardiopulmonary Sequelae

COVID-19 can cause cardiopulmonary sequelae in patients with serious infection requiring hospitalization, which may lead to persisting symptoms (fatigue, dyspnea, chest pain, cough) and alterations in diagnostic tests that persist after acute infection has healed.

- Pulmonary sequelae (residual post-pneumonia interstitial involvement). Studies have reported that a significant percentage of patients show abnormal results in respiratory function (54%) and CT imaging studies (40–94%) one month after infection [33,48,49], and fibrosis has been detected in 26% at three months (50% in patients requiring ICU admission) [50].
- Pleural involvement has also been linked to acute COVID-19 infection, with an estimated frequency of pleural thickening of 27% and of pleural effusion of 5–6%. There are no data on chronicity [51,52].
- Cardiac involvement. Myocarditis in COVID-19 patients occurs mainly during the first two weeks, although several cases presenting some weeks after resolution of the infection are reported [26]. Studies show that, at about two months post-diagnosis, 40–80% of patients may have increased troponin I levels and 78% cardiac involvement on cardiac MRI. The clinical significance in asymptomatic patients remains unclear [53,54].
- Pericardial effusion has been reported in 5% of COVID-19 patients, especially in those with myocarditis, and cardiac tamponade in 1% of hospitalized patients [52]. More than one in three previously-healthy college athletes recovering from COVID-19 infection showed resolving pericardial inflammation on imaging [55].

In these patients, multidisciplinary care is needed to coordinate primary and hospital care.

#### 6.1.2. Post-COVID-19 Thrombosis

Some patients with COVID-19 may develop thrombosis once the acute infection is resolved. Although COVID-19 infection has been linked to coagulopathy, especially in severe patients admitted to the ICU, SARS-CoV-2 does not appear to have intrinsic prothrombotic effects, and commonly detected coagulation test abnormalities in COVID-19 patients appear to be primarily related to the systemic inflammatory response, with frequent detection of antiphospholipid antibodies (especially lupus anticoagulant) in severe patients, but with no clear correlation between positivity and thrombosis [26]. The global risk of venous thromboembolism after hospital discharge is estimated at 3% of patients (up to 80% of events at 30–45 days), while the reported frequency of post-discharge thrombosis in COVID-19 patients is 0.5–2.5% [56,57], similar to or lower than that described in non-COVID-19 populations. If vascular ultrasound is not available in primary care, application of the Wells criteria remains useful.

#### 6.1.3. Post-COVID-19 Immune-Mediated Manifestations

Many of the broad spectrum of post-COVID-19 immune-mediated manifestations may occur occasionally in the post-infectious phase [26]. Most are very infrequent and have an autoimmune basis. Despite this, primary care physicians should be aware of these manifestations, as early suspicion and guidance are crucial in most cases. The main ones are:

- Arthritis: If there are inflammatory data on the physical examination or ultrasound scan, post-COVID-19 arthritis should be ruled out. Fewer than 10 cases are reported worldwide, affecting men, with a mean age of 54 years and a variety of joint presentations (symmetrical polyarthritis, monoarthritis, enthesitis, and psoriatic arthritis), and mainly appear once the infection is resolved [26].
- Myositis: The frequency of elevated creatine kinase levels in acute COVID-19 infection is approximately 10%, but there are no data on persistence. A review of isolated cases of COVID-19 patients with myositis/rhabdomyolysis showed most cases occurred in adult males with myalgia (in some cases severe) and appeared mainly during the first week of COVID-19 infection, with creatine kinase levels >10,000 U/L and renal impairment [26].
- Pancreatitis: COVID-19 patients with abdominal pain and elevated pancreatic enzymes diagnosed with acute pancreatitis (mostly females) are reported. The clinical and epidemiological scenario is broad and includes infants and older patients, patients with clinical symptoms, post-mortem studies, familial cases, and patients with underlying predisposing factors [26].
- Other manifestations: Skin (perniosis), neurological (encephalitis, Guillain-Barré syndrome, myelitis), renal (tubulopathies, glomerulonephritis), hematological (idiopathic thrombocytopenic purpura, autoimmune hemolytic anemia), endocrine (thyroiditis, manifesting as clinical symptoms of thyrotoxicosis), and systemic autoimmune (lupus, vasculitis, sarcoidosis, Kawasaki disease) diseases have been reported in COVID-19 patients [26].

### 6.2. Specific Evaluation of Emotional Well-Being and Mental Health

The impact that the pandemic has produced on mental health is still far from being adequately measured and quantified in primary care. In patients who have contracted COVID-19, the impact is undeniable, although, etiopathogenically, it is more reasonable to consider it a reactive manifestation after experiencing a highly traumatic situation (with severe implications for the affected person, not only with respect to their own health, but also with deep family, social, and work implications) that do not classify it as a manifestation directly caused by the virus. Despite this, the importance of these manifestations requires a specific section, not only because of their enormous impact on the quality of life of the affected person, but also because of the possible impact they may have on their relationship with other manifestations of long COVID-19.

A key function of primary care is not to medicalize these situations, always considering the key role of social determinants such as poverty, discrimination, and social exclusion; mental health and well-being are reinforced by increased social solidarity, informal social support, mutual assistance and other collective or community-based measures. Most patients infected by the virus did not required hospital admission and were diagnosed and followed in primary care, but isolation leads to reduced physical activity and greater isolation, which may be particularly problematic in older adults, due to reduced physical ability and potential increases in mental health problems, such as anxiety and depression. Persons with previous psychiatric disease who have experienced worsening or decompensation during COVID-19 disease are especially vulnerable. Isolation during hospital admission can also lead to a worse experience of the pandemic disease process. Patients have experienced new anti-contagion preventive standards, including isolation in their rooms, distancing at stressful vital moments (such as serious illness or a poor prognosis) which may sometimes have an impact on the human relationship with the doctor. COVID-19 survivors after ICU admission have an increased risk of long-lasting severe functional limitations, psychological distress, post-traumatic stress disorder and depression [58]. A recent study shows that 36% of patients had altered mental and cognitive questionnaire scores (HADS, TICS, CFQ, PCL-5, IES-R) at three months, although there were no significant differences between patients according to COVID-19 severity [59].

### 6.3. Diagnosis of Post-COVID-19 Syndrome

After ruling out the above-mentioned processes, a tentative diagnosis of ongoing COVID-19 in the second visit (V2) may be made, enabling the primary care team plan for V3, which will evaluate the evolution (whether symptoms still persist) and reassess possible causes of the long-term symptoms using the corresponding diagnostic algorithms; if the symptoms persist and no etiology reasonably explains the persisting symptoms and complementary tests at V3 are unaltered, the diagnosis of post-COVID-19 syndrome is confirmed, and a diagnostic approach to some post-COVID-19 syndromic presentations should be made:

Long-term fatigue: If fatigue persists as the main symptom, compliance with the classification criteria for chronic fatigue syndrome should be evaluated. If the criteria are met, the diagnosis of chronic fatigue syndrome associated with COVID-19 (which will be confirmed when the criteria are still fulfilled at six months) is made and the protocol of referral to the corresponding multidisciplinary unit is applied. In the case of non-compliance, evaluation of the loss of physical condition related to the pandemic and psychological factors is recommended, as are adapted guidelines to increase progressive resistance training, physical activity programs, etc.

Long-term generalized pain: When generalized pain persists as the main symptom, compliance with the classification criteria for chronic generalized pain (CGP) and fibromyalgia (FM) should be evaluated. If positive, the diagnosis of CGP or FM associated with COVID-19 is established and the patient should be referred to the pain unit or rheumatology department, depending on availability or closeness. Other factors (loss of physical condition, psychological factors) should be evaluated if not.

Long-term dyspnea: When dyspnea persists as the main symptom, and after ruling out all complementary tests for alternative diseases, the so-called respiratory burn syndrome, or chronic inflammation of the respiratory tract (trachea, bronchi, bronchioles) may be hypothesized, and referral to pulmonology or otolaryngology considered.

Long-term anosmia/dysgeusia: In these patients, referral to otolaryngology for follow-up and treatment using specific olfactory training therapies is recommended.

Long-term headache: There are no studies on the characteristics of long-term headache in COVID-19, but it could be included among the primary headaches, which are diagnosed according to symptoms in the absence of organic or structural alterations. Ruling out a central sensitization syndrome, and a possible neurology consultation are recommended.

Long-term digestive signs and symptoms: In cases of chronicity (duration >3 months), ruling out central sensitization syndromes and evaluating referral to the digestive department are recommended.

## 7. Limitations and Perspective

The lack of a scientifically accepted definition, together with the limited currently-available scientific evidence and the wide methodological differences among the few reported studies, make it very difficult to assess the frequency of long COVID-19. Studies of symptomatic COVID-19 at >4 weeks post-diagnosis have widely-differing methodologies and no overall figure can be suggested, especially in outpatients followed in primary care. Studies suggest about 10% of COVID-19 patients may have related symptoms beyond 3 weeks, which persist in some patients for months [24,25], while the UK Office for National Statistics has estimated that 20% may have symptoms that persist after 5 weeks, and 10% may have symptoms for 12 weeks or longer after acute infection [60]. The largest study until now has evaluated symptoms in >4000 people through a mobile application, reporting figures of 13.3% at four weeks, 4.5% at eight weeks and 2.3% at 12 weeks [20]. In contrast, a recent Chinese study in 1655 patients with COVID-19 who required hospitalization has shown that 76% of patients reported ≥1 symptom at six months of follow-up (measured using a self-reported symptom questionnaire asking about newly-occurring persistent symptoms or any symptoms that worsened after COVID-19), of which 63% were associated with fatigue or muscle weakness [61]. This very wide variation in the frequency of long COVID-19 symptoms between studies is also reported with respect to the individual frequency of the main individual symptoms eight weeks after acute infection [28,29,30,31,32,33,34,37,40,41,42,43,44,47] (Table 3). Unfortunately, there are wide variations in study designs, populations evaluated (unselected, or specifically studied in a particular specialty or pathology) and symptom identification (self-report, medical evaluation with or without tests), and there is also the lack of a standardized definition of long-term symptoms, since aggravated prior symptoms or symptoms derived from the typical aftermath of severe bilateral pneumonia may be included. Progress cannot be made without a minimum consensus on the definition of the variables to be measured (the timing of the appearance of long-term symptoms -before, during or after acute infection- should be clearly defined) and the measurement instruments (usually unvalidated, self-reported questionnaires, some measured dichotomously, others categorized, others quantified with VAS scales). Another significant limitation, as already seen in some studies [62], is the measurement of the gross frequency at a specific time point with respect to the total number of patients, allowing the inclusion of people who develop the symptom de novo once the acute infection is resolved. This is very important when assessing symptoms of general involvement (fatigue, chronic pain, headache, memory disturbances...) that may be more closely related to psychological and social determinants than to a direct etiopathogenic link with SARS-CoV-2 infection.

Little is known about the etiopathogenic mechanisms responsible for symptom persistence in COVID-19. Since long-term manifestations affect various organs and systems, they may have very diverse etiopathogenic origins, probably driven by genetic predisposition, some pathological mechanisms of the virus, the phenotypic presentation of the disease in acute infections, and the individual immune response. Studies detecting viral RNA persistence in respiratory and extra-respiratory tissues weeks after acute infection are increasingly common, although a clear pathogenic link between viral detection and virus-related organ-specific damage is remains unclear [63,64,65,66,67]. Some studies have suggested potential immune-mediated mechanisms of damage, e.g., in patients with migraine-like features, activation of the trigeminovascular system by inflammation or direct SARS-CoV-2 involvement has been suggested [45], while structural alterations (olfactory cleft and olfactory bulb abnormalities on MRI) have been identified in patients presenting with long-term anosmia [68].

**Figure 1:**
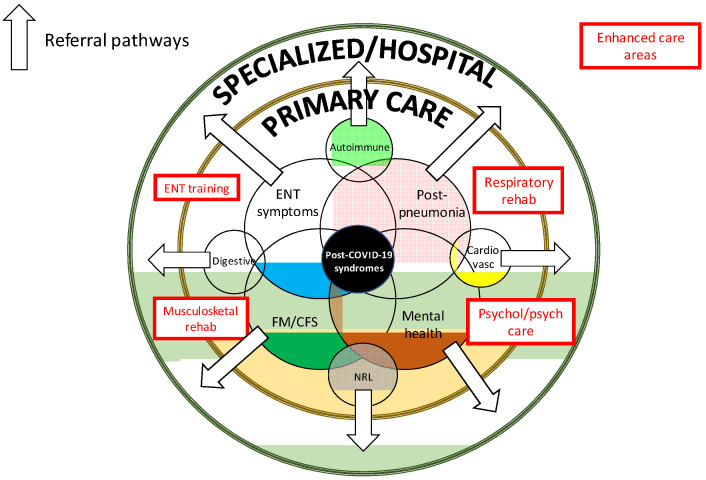
A graphic proposal for the multidisciplinary care of patients with long COVID-19 in primary care. Rehab: rehabilitation, ENT: Ear, Nose and Throat, FM: fibromyalgia, CFS: chronic fatigue syndrome, NRL: neurological, Cardiovasc: cardiovascular, Psychol/psych: psychological/psychiatric.

By organ, the main long-term manifestations observed in other coronaviruses (SARS, MERS) have very clear pathological parallels with SARS-CoV-2: mainly respiratory, musculoskeletal and neuropsychiatric [24,25]. Structuring this wide variety of symptoms and alterations that some COVID-19 patients may present after recovery from the infection into syndromes that can reasonably group them medically for better management and identification is a challenge. Some opinion articles have suggested differentiated syndromes relating to some symptoms or groups of prominent symptoms, such as general symptoms around fatigue, ENT symptoms, severe post-pneumonia respiratory consequences or mental health [69] (Figure 1). Among the signs and symptoms reported, fatigue is undeniably the key symptomatic marker of long COVID-19, both in frequency and the impact on the quality of life [60], and is linked to a wide variety of concomitant systemic symptoms (diffuse myalgia, asthenia, depression, sleep disturbances…) that are similar to post-SARS and other post-viral syndromes [70,71] and with a syndromic profile overlapping with central sensitivity syndromes such as CFS and FM. Several epidemiological and socioeconomic factors directly associated with the pandemic have been implicated in the pathogenesis of these processes, including distress, insomnia, reduced physical activity and changes in diet, and lifestyle related to confinement [24,25]. Various neuropsychological alterations related to memory, sleep quality, or concentration presented by patients with long-COVID-19 are also part of central sensitization syndromes. Although most patient associations are opposed to long COVID-19 being compared with or assimilated to other syndromes characterized by a set of subjective symptoms in the absence of objective alterations in diagnostic examinations [60], the overlap of the signs and symptoms they present with processes like fibromyalgia, chronic fatigue syndrome, or multiple chemical sensitivity is, medically, incontestable.

Information on factors that may identify populations most at risk of long COVID-19 is very scarce. A prepress study suggests an increased risk of long COVID-19 in females, persons aged >70 years, and patients requiring hospitalization during the acute infection, and asthma, without differences between countries or socioeconomic groups. The number of symptoms during the acute infection also appears to have an influence, as people with ≥5 symptoms more often had long-term symptoms. The symptoms with the greatest predictive power were fatigue, headache, dyspnea, hoarseness and myalgia (in people aged ≥70 years: fever, loss of smell and cardiopulmonary comorbidities) [20]. Another study showed a higher frequency in people aged ≥50 years but no other risk factors [59], and a higher risk of long-term anxiety/depression and fatigue/muscle weakness in women and older patients [61]. The impact on the quality of life is multidimensional but appears to be related to the care received according to severity as, in patients who required ICU admission, the worsened quality of life focused especially on pain and mobility while, in non-hospitalized patients, it focused on anxiety or depression [34].

Not surprisingly, considering the lack of reasonable pathogenic mechanisms involved in long COVID-19, there is no clear treatment pathway. We endorse the key points for managing long COVID-19 proposed by the NICE guidelines [23], including giving advice and information on self-management of symptoms, self-monitoring at home (heart rate, blood pressure, pulse oximetry, sleep surveillance), a central role for multidisciplinary rehabilitation support (covering physical, psychological and psychiatric aspects) with occupational therapy, physiotherapy, clinical psychology and psychiatric therapy and rehabilitation medicine (Figure 1).

Unfortunately, the current level of scientific evidence on long COVID-19 is very low, and the lack of internationally accepted definitions results in such a high level of heterogeneity that it makes overall analysis and, even more, meta-analysis, very difficult. As strong conclusions and plausible etiopathogenic explanations are not available, the main objective of these guidelines is to arouse scientific interest, thus facilitating studies of the pathogenic mechanisms that may aid the early detection and correct management of long COVID-19 manifestations.

## 8. Conclusions

Long-term manifestations are increasingly recognized in COVID-19 patients, with systemic clinical presentations affecting a wide range of organs and systems. However, the natural history of long COVID-19 in primary care remains unknown. The estimated 10% of COVID-19 survivors with a long-term course translates into 200,000 people in Spain, with the number increasing daily. Patients, many young and healthy before their illness, report in the press and on social media worldwide that health professionals reject them or treat them as hypochondriacs [24,25,60]. Based on the current limited evidence, a significant percentage of patients with long COVID-19 are expected to recover without the need for hospital care, so a holistic, longitudinal primary care follow up will suffice, with the involvement of multidisciplinary rehabilitation services and the creation of groups of affected patients from the same community or territory that harness the potential of the use of videos and other technologies remotely. Long COVID-19 should be managed jointly with pre-existing comorbidities and those caused by the infection. We are aware of the limitations that a first guideline proposal in primary care may have, and that continuous updates are essential, as some editorials pointed out for the first guidelines published by NICE [60,72]. Considering the very limited evidence available at the time of writing, we consider these guidelines as ‘living’ guidelines that will be periodically reviewed and updated, as most of the keys to ensuring adequate management of long COVID-19 remain to be defined (frequency, natural history, risk factors, prognostic markers, validated tools, interventions) [23].

However, the volume of current and future long COVID-19 patients is so high that primary care may not be able to cope with their care, considering current resources and the protocolized care of post-COVID-19 patients in primary care. The feasibility of the application of this CPG would only be possible with the provision of additional, specific human resources to serve this large group of patients. In addition, if comprehensive multidisciplinary care is really to be implemented, it is also essential to provide primary care centers with additional support staff in the main areas identified (rehabilitation, mental health) trying to avoid fragmented care. Any attempt to add this care to the primary care portfolio of services with the human resources currently available would result in a resounding and absolute failure.

## Figures and Tables

**Table 1:** Frequency of the main signs and symptoms of acute SARS-CoV-2 infection described in publications with representative cohorts of more than 1000 patients) [5,6,7,8,9,10,11,12,13,14,15,16,17,18,19].

	Signs and Symptoms	Frequency (*n/N*)	Percentage	Studies (*n*)
Respiratory	Cough	107,044/135,767	78.8	15
	Dyspnea	71,604/166,030	43.1	14
	Expectoration	12,383/66,211	18.7	10
	Chest pain	9603/71,793	13.4	6
Constitutional	Fever	123,188/168,346	73.2	16
	Fatigue	60,006/144,955	41.4	12
	Chills/shivers	7244/60,661	11.9	5
	Wheezing	5109/63,937	8.0	2
	Syncope	53/1841	2.9	2
	Edema	30/1968	1.5	2
Rheumatic *	Myalgia	15,337/76,919	19.9	13
	Myalgia and/or arthralgia	8277/55,924	14.8	1
	Arthralgia	4619/61,675	7.5	3
Otolaryngological	Sore throat	14,252/123,319	11.6	9
	Dysgeusia	3483/38,484	9.1	5
	Anosmia	4494/56,356	8.0	7
	Rhinorrhea	3519/65,987	5.3	7
	Nasal congestion	2684/55,924	4.8	1
	Hemoptysis	660/61,775	1.1	6
	Otalgia	631/75,336	0.8	2
Digestive complaints	Anorexia	4084/19,092	21.4	4
	Diarrhea	20,249/153,778	13.2	13
	Nausea or vomiting	17,142/136,902	12.5	13
	Abdominal pain	7421/69,573	10.7	4
Neurological	Confusion/altered consciousness	18,434/70,032	26.3	2
	Headache	17,734/128,233	13.8	12
Other	Conjunctivitis	782/138,724	0.6	5

* Among the different categories of signs and symptoms, there is a wide heterogeneity of definition of each variable between the different studies, and therefore, the frequencies derived from merging data from different studies should be interpreted with caution.

**Table 2:** Comprehensive evaluation of long COVID-19. (**a**). Laboratory tests. (**b**). Other diagnostic tests. Tests for each symptom are not mandatory but depend on individualized medical assessment.

(a)										
LABORATORY TESTS	Fatigue	Arthralgia	Myalgia	Chest Pain	Cough	Dyspnea	Anosmia	Dysgeusia	Headache	Digestive Complaints
Hemogram	+	+	+	+	+	+	+	+	+	+
c-reactive protein/erythrocyte sedimentation rate/ferritin	+	+	+	+	+	+	+	+	+	+
D-Dimer	+	+	+	+	+	+	+	+	+	+
Na/K	+	+	+	+	+	+	+	+	+	+
Liver profile	+	+	+	+	+	+	+	+	+	+
Renal profile	+	+	+	+	+	+	+	+	+	+
Thyroid function	+	+	+	+	+	+	+	+	+	+
Proteinogram	+	+	+	+	+	+	+	+	+	+
Nutritional profile	+									+
Pancreatric profile				+						+
Natriuretic peptides				+		+				
Muscular enzymes			+	+		+				
Serum cortisol	+									
Rheumatoid factor/antinuclear antibodies/complement		+	+							
Anti-transglutaminase antibodies										+
**(b)**										
**OTHER DIAGNOSTIC TESTS**	**Fatigue**	**Arthralgia**	**Myalgia**	**Chest Pain**	**Cough**	**Dyspnea**	**Anosmia**	**Dysgeusia**	**Headache**	**Digestive Complaints**
Vital signs	+	+	+	+	+	+	+	+	+	+
Oxygen saturation	+	+	+	+	+	+	+	+	+	+
Electrocardiogram	+	+	+	+	+	+	+	+	+	+
Chest X-ray/lung ultrasound	+	+	+	+	+	+	+	+	+	+
Spirometry	+			+	+	+				
Chest computed tomography				+		+				
Funduscopy									+	
Joint ultrasound		+								
Abdominal ultrasound										+
Fecal occult blood										+
Digestive endoscopy										+

**Table 3:** Summary of data from the main studies on symptoms reported as long COVID-19 (>4 w) * [28,29,30,31,32,33,34,37,40,41,42,43,44,47].

Signs and Symptoms of Long COVID-19		Weeks after the First Symptom of Acute COVID-19 Infection		
		4 w	8 w	12 w
**Global frequency**		13.3%	4.5%	2.3%
**Constitutional**	Fever	-	0% [37], 3% [29]	-
	Chills	5% [28]	-	-
	Fatigue	35% [28], 45% [29]	30% [30], 53% [31], 77% [32]	16% [33], 55% [34]
**Rheumatic manifestations**	Arthralgia	10% [37], 15% [28]	16% [37], 27% [31]	-
	Myalgia	15% [29]	6% [31], 13% [30]	16% [34]
**Respiratory manifestations**	Dyspnea	11% [37], 27% [28], 33% [29]	8% [37], 31% [30], 43% [31], 63% [32]	14% [33]
	Chest pain	20% [28]	22% [31]	11% [34]
	Cough	33% [29], 43% [28]	5% [30], 18% [31], 46% [32]	2% [33], 17% [34]
	Expectoration	-	8% [31]	2% [33]
**Otolaryngological manifestations**	Rhinorrhea	28% [28]	12% [29], 15% [31]	-
	Sore throat	15% [28]	7% [31], 9% [29]	-
	Anosmia	12% [29], 23% [28], 28% [40], 43% [41], 46% [42], 56% [43]	2% [30], 17% [31], 25% [42]	13% [34], 46% [44]
	Dysgeusia	9% [29], 17% [40], 24% [28], 50% [43]	1% [30], 10% [31]	11% [34], 31% [44]
	Anosmia/Dysgeusia	28% [37], 9% [47]	2% [47], 23% [37]	4% [33]
**Digestive complaints**	Abdominal pain	15% [28]	3% [29]	-
	Nausea	10% [28]	6% [29]	-
	Vomiting	4% [28]	-	-
	Diarrhea	-	3% [31], 9% [29]	-
	Diarrhea or vomiting	17% [37]	11% [37]	31% [33]
	Anorexia	-	8% [31]	-
	Weight loss >5%	16% [37]	17% [37]	-
**Neurological manifestations**	Headache	14% [28]	9% [31], 15% [29]	18% [33]
	Behavioral disorder	-	-	27% [34]
	Memory loss	-	-	34% [34]
	Sleep disorders	-	-	31% [34]
	Vertigo/dizziness	-	6% [31]	-
**Other manifestations**	Dry syndrome	-	16% [31]	-
	Hair loss	-	-	20% [34]
	Conjunctivitis	-	16% [31]	-

* Unfortunately, there are wide variations in study designs, populations evaluated (unselected, or specifically studied in a particular specialty or pathology) and in the collection of symptoms (self-report, or medical evaluation with or without scans), and a lack of standardized definitions of persistent symptoms, since aggravated prior symptoms or symptoms derived from the aftermath of severe bilateral pneumonia may be included.

## Data Availability

No new data were created or analyzed in this study. Data sharing is not applicable to this article.

## Supplementary Materials

The following are available online at https://www.mdpi.com/article/10.3390/ijerph18084350/s1, File S1: Algorithms S1–S9.
