# Supplementary material for: Long Covid-19: Proposed Primary Care Clinical Guidelines for Diagnosis and Disease Management
Source: Int J Environ Res Public Health. 2021 Apr 20;18(8):4350. doi: 10.3390/ijerph18084350 (PMC8073248; doi:10.3390/ijerph18084350)
Supplement: Supplementary file 1 [file ijerph-18-04350-s001.zip › ijerph-1135204-SI.pdf]

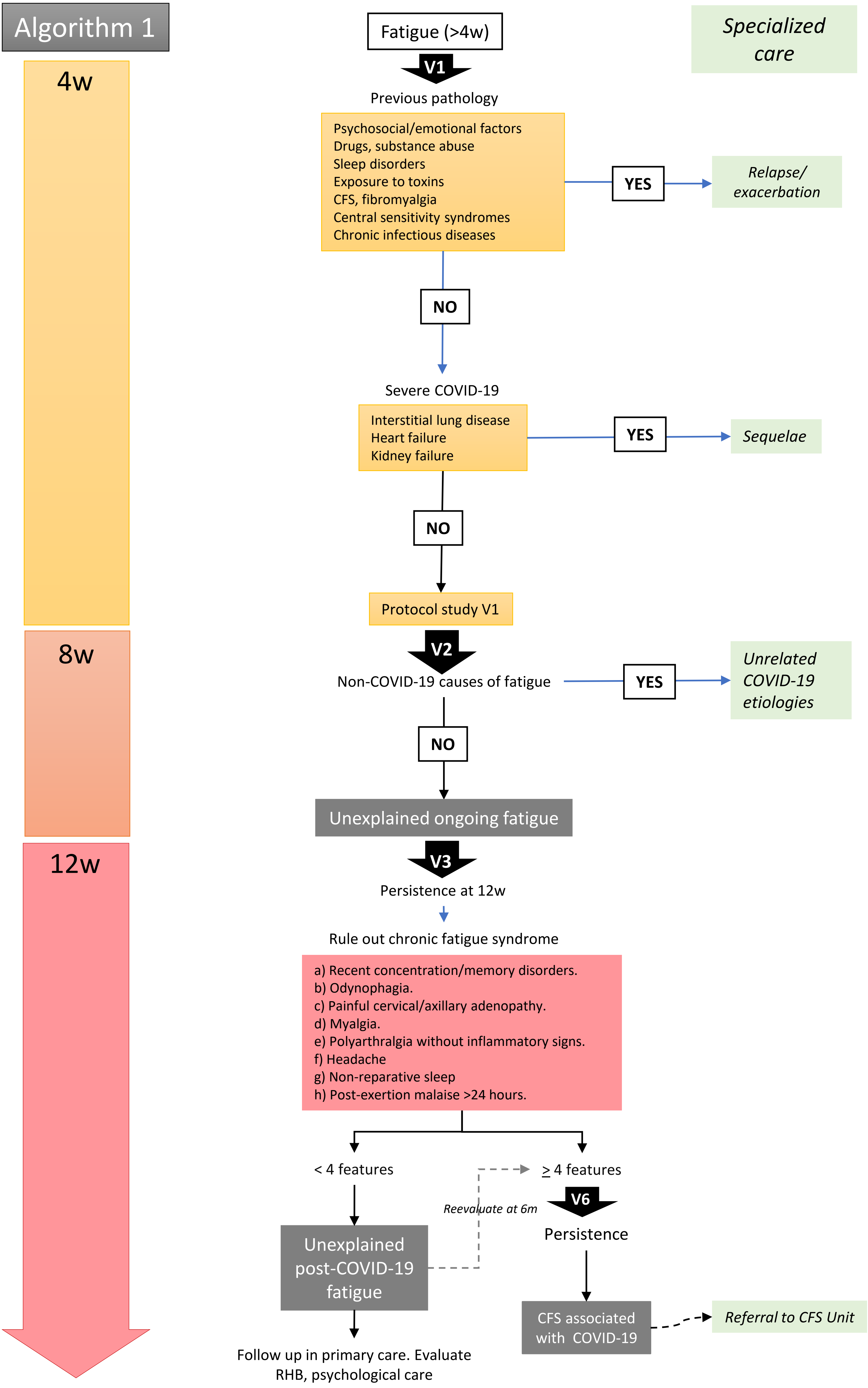

Algorithm 1
Specialized care
4w
8w
12w
Fatigue (>4w)
V1
Previous pathology
Psychosocial/emotional factors
Drugs, substance abuse
Sleep disorders
Exposure to toxins
CFS, fibromyalgia
Central sensitivity syndromes
Chronic infectious diseases
YES
Relapse/ exacerbation
NO
Severe COVID-19
Interstitial lung disease
Heart failure
Kidney failure
YES
Sequelae
NO
Protocol study V1
V2
Non-COVID-19 causes of fatigue
YES
Unrelated COVID-19 etiologies
NO
Unexplained ongoing fatigue
V3
Persistence at 12w
Rule out chronic fatigue syndrome
a) Recent concentration/memory disorders.
b) Odynophagia.
c) Painful cervical/axillary adenopathy.
d) Myalgia.
e) Polyarthralgia without inflammatory signs.
f) Headache
g) Non-reparative sleep
h) Post-exertion malaise >24 hours.
< 4 features
≥ 4 features
Reevaluate at 6m
V6
Persistence
Unexplained post-COVID-19 fatigue
CFS associated with COVID-19
Referral to CFS Unit
Follow up in primary care. Evaluate RHB, psychological care

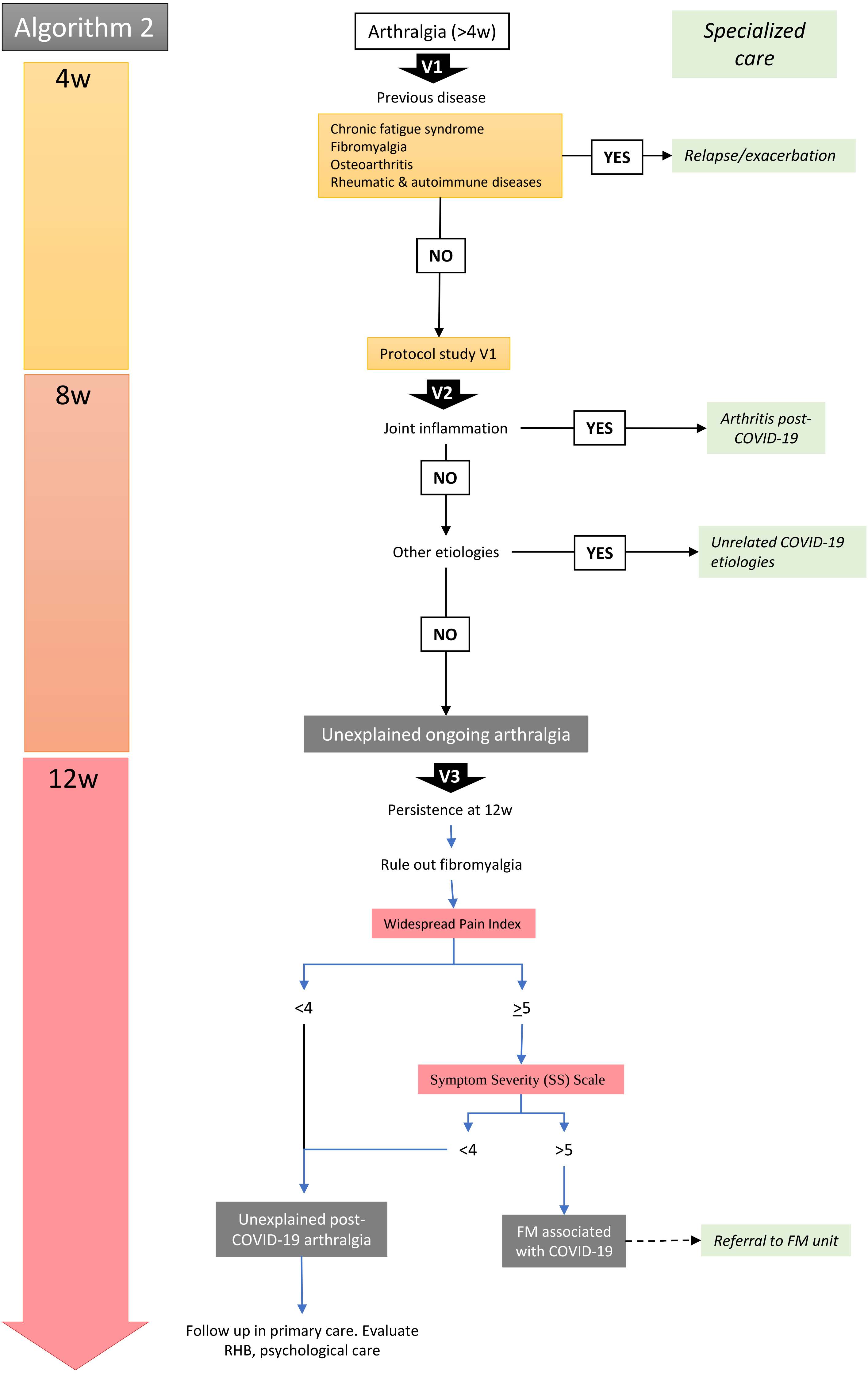
Algorithm 2
4w
8w
12w
Arthralgia (>4w)
Specialized care
V1
Previous disease
Chronic fatigue syndrome
Fibromyalgia
Osteoarthritis
Rheumatic & autoimmune diseases
YES
Relapse/exacerbation
NO
Protocol study V1
V2
Joint inflammation
YES
Arthritis post-COVID-19
NO
Other etiologies
YES
Unrelated COVID-19 etiologies
NO
Unexplained ongoing arthralgia
V3
Persistence at 12w
Rule out fibromyalgia
Widespread Pain Index
<4
≥5
Symptom Severity (SS) Scale
<4
>5
Unexplained post-COVID-19 arthralgia
FM associated with COVID-19
Referral to FM unit
Follow up in primary care. Evaluate RHB, psychological care

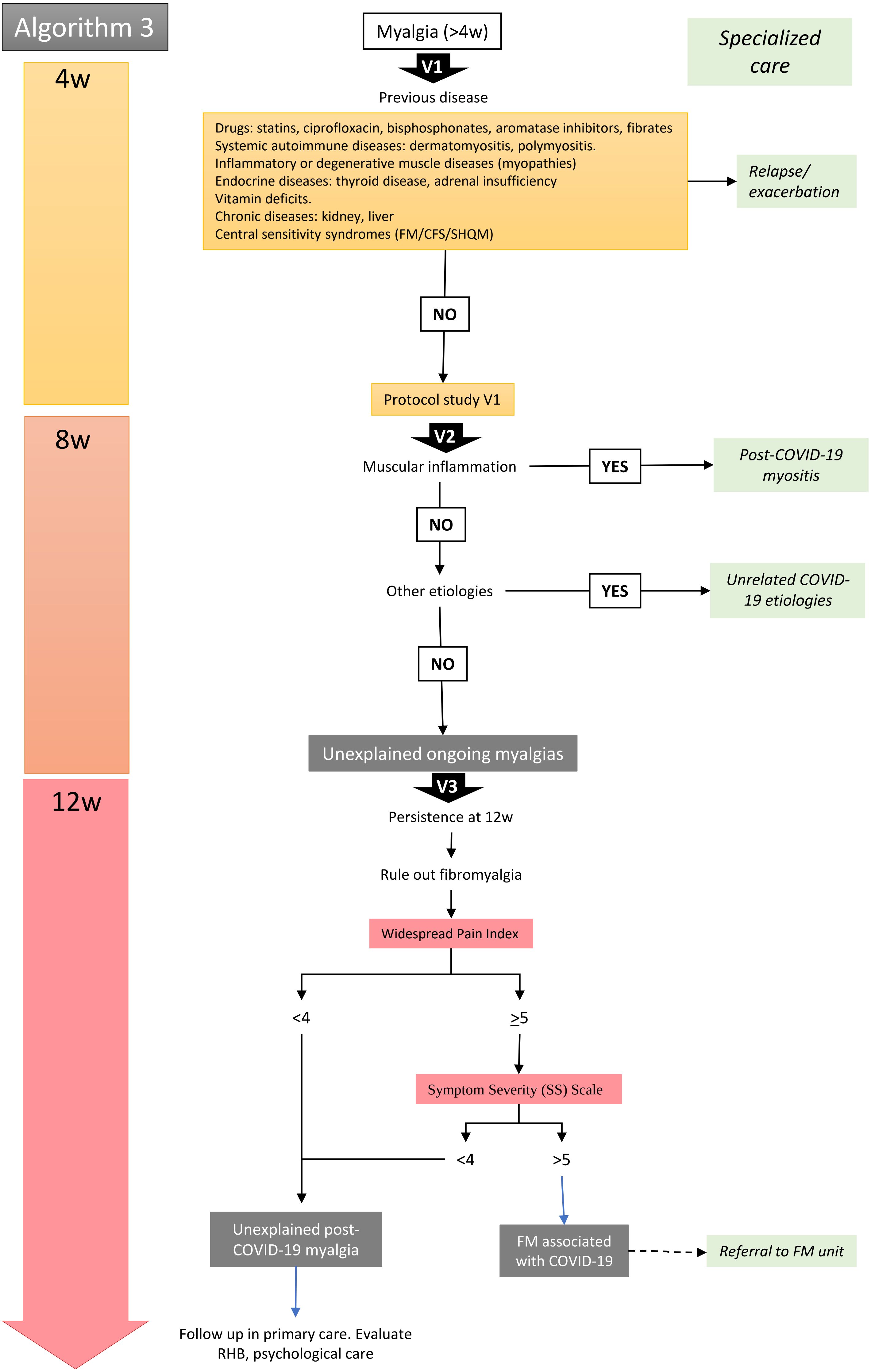

Algorithm 3
4w
8w
12w
Myalgia (>4w)
V1
Previous disease
Drugs: statins, ciprofloxacin, bisphosphonates, aromatase inhibitors, fibrates
Systemic autoimmune diseases: dermatomyositis, polymyositis.
Inflammatory or degenerative muscle diseases (myopathies)
Endocrine diseases: thyroid disease, adrenal insufficiency
Vitamin deficits.
Chronic diseases: kidney, liver
Central sensitivity syndromes (FM/CFS/SHQM)
Specialized care
Relapse/ exacerbation
NO
Protocol study V1
V2
Muscular inflammation
YES
Post-COVID-19 myositis
NO
Other etiologies
YES
Unrelated COVID-19 etiologies
NO
Unexplained ongoing myalgias
V3
Persistence at 12w
Rule out fibromyalgia
Widespread Pain Index
<4
≥5
Symptom Severity (SS) Scale
<4
>5
Unexplained post-COVID-19 myalgia
FM associated with COVID-19
Referral to FM unit
Follow up in primary care. Evaluate RHB, psychological care

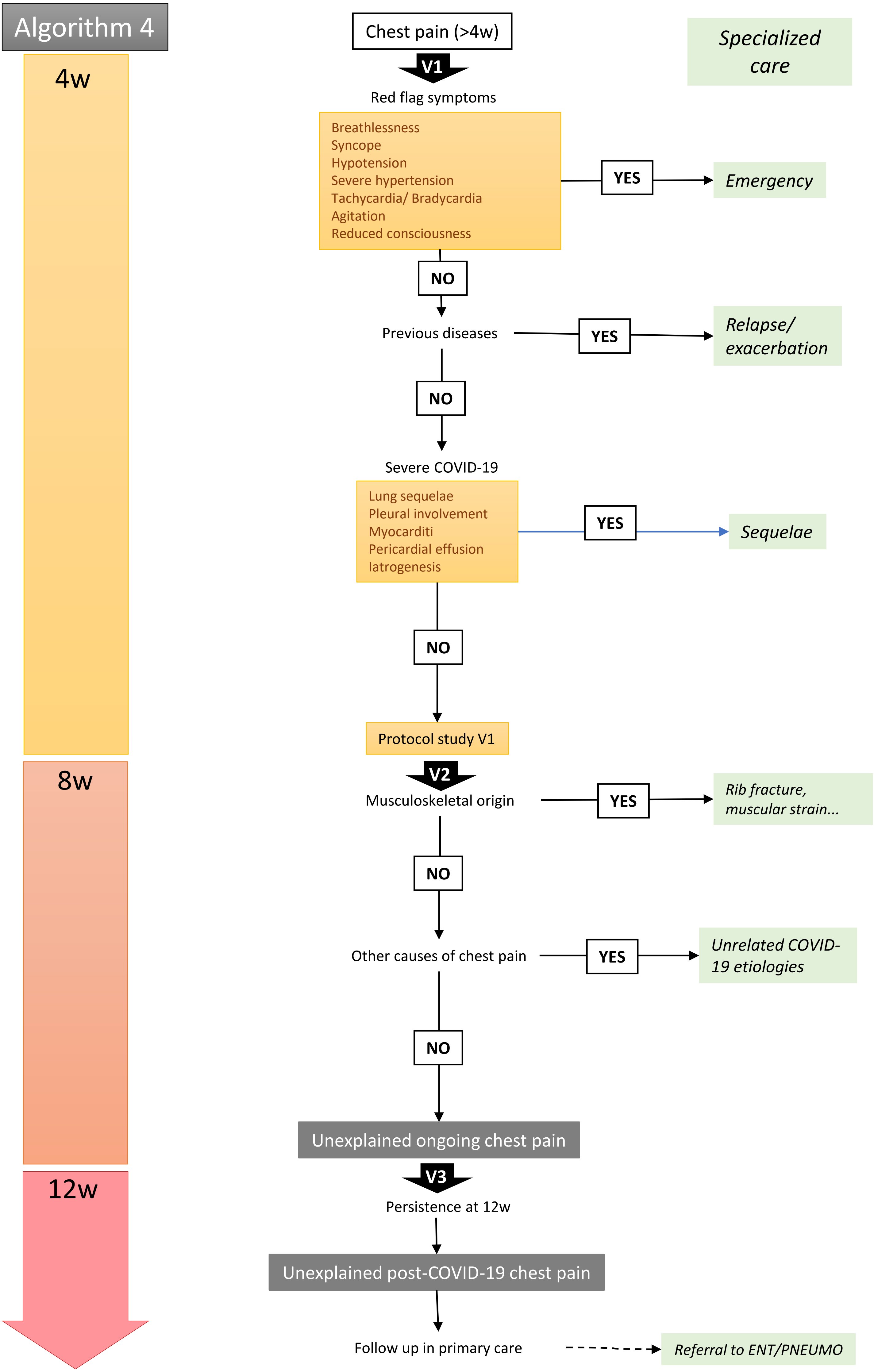

Algorithm 4
4w
8w
12w
Chest pain (>4w)
V1
Red flag symptoms
Breathlessness
Syncope
Hypotension
Severe hypertension
Tachycardia/ Bradycardia
Agitation
Reduced consciousness
YES
Emergency
NO
Previous diseases
YES
Relapse/ exacerbation
NO
Severe COVID-19
Lung sequelae
Pleural involvement
Myocarditi
Pericardial effusion
Iatrogenesis
YES
Sequelae
NO
Protocol study V1
V2
Musculoskeletal origin
YES
Rib fracture, muscular strain...
NO
Other causes of chest pain
YES
Unrelated COVID-19 etiologies
NO
Unexplained ongoing chest pain
V3
Persistence at 12w
Unexplained post-COVID-19 chest pain
Follow up in primary care
Referral to ENT/PNEUMO
Specialized care

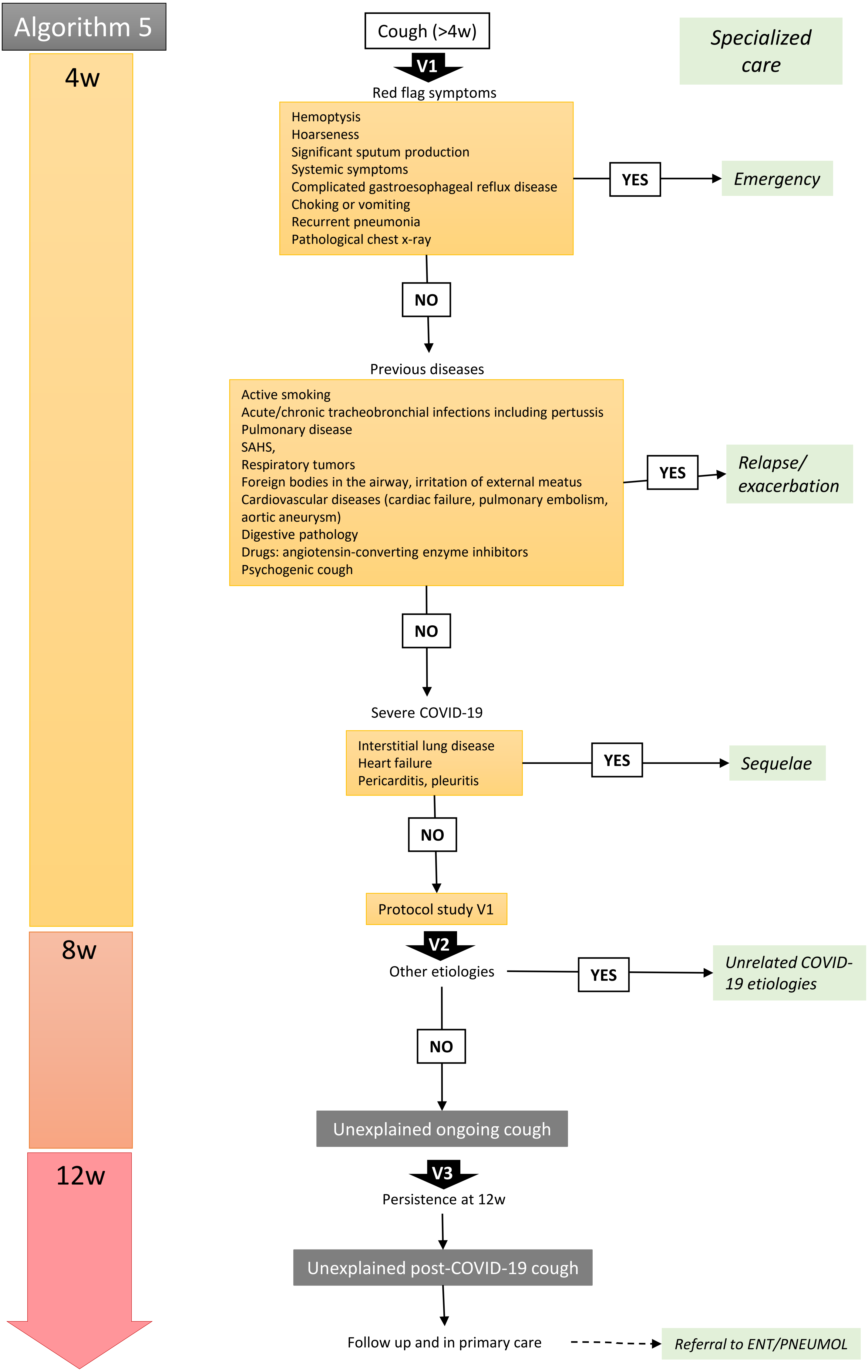
Algorithm 5
4w
8w
12w
Cough (>4w)
Specialized care
V1
Red flag symptoms
Hemoptysis
Hoarseness
Significant sputum production
Systemic symptoms
Complicated gastroesophageal reflux disease
Choking or vomiting
Recurrent pneumonia
Pathological chest x-ray
YES
Emergency
NO
Previous diseases
Active smoking
Acute/chronic tracheobronchial infections including pertussis
Pulmonary disease
SAHS,
Respiratory tumors
Foreign bodies in the airway, irritation of external meatus
Cardiovascular diseases (cardiac failure, pulmonary embolism, aortic aneurysm)
Digestive pathology
Drugs: angiotensin-converting enzyme inhibitors
Psychogenic cough
YES
Relapse/ exacerbation
NO
Severe COVID-19
Interstitial lung disease
Heart failure
Pericarditis, pleuritis
YES
Sequelae
NO
Protocol study V1
V2
Other etiologies
YES
Unrelated COVID-19 etiologies
NO
Unexplained ongoing cough
V3
Persistence at 12w
Unexplained post-COVID-19 cough
Follow up and in primary care
Referral to ENT/PNEUMOL

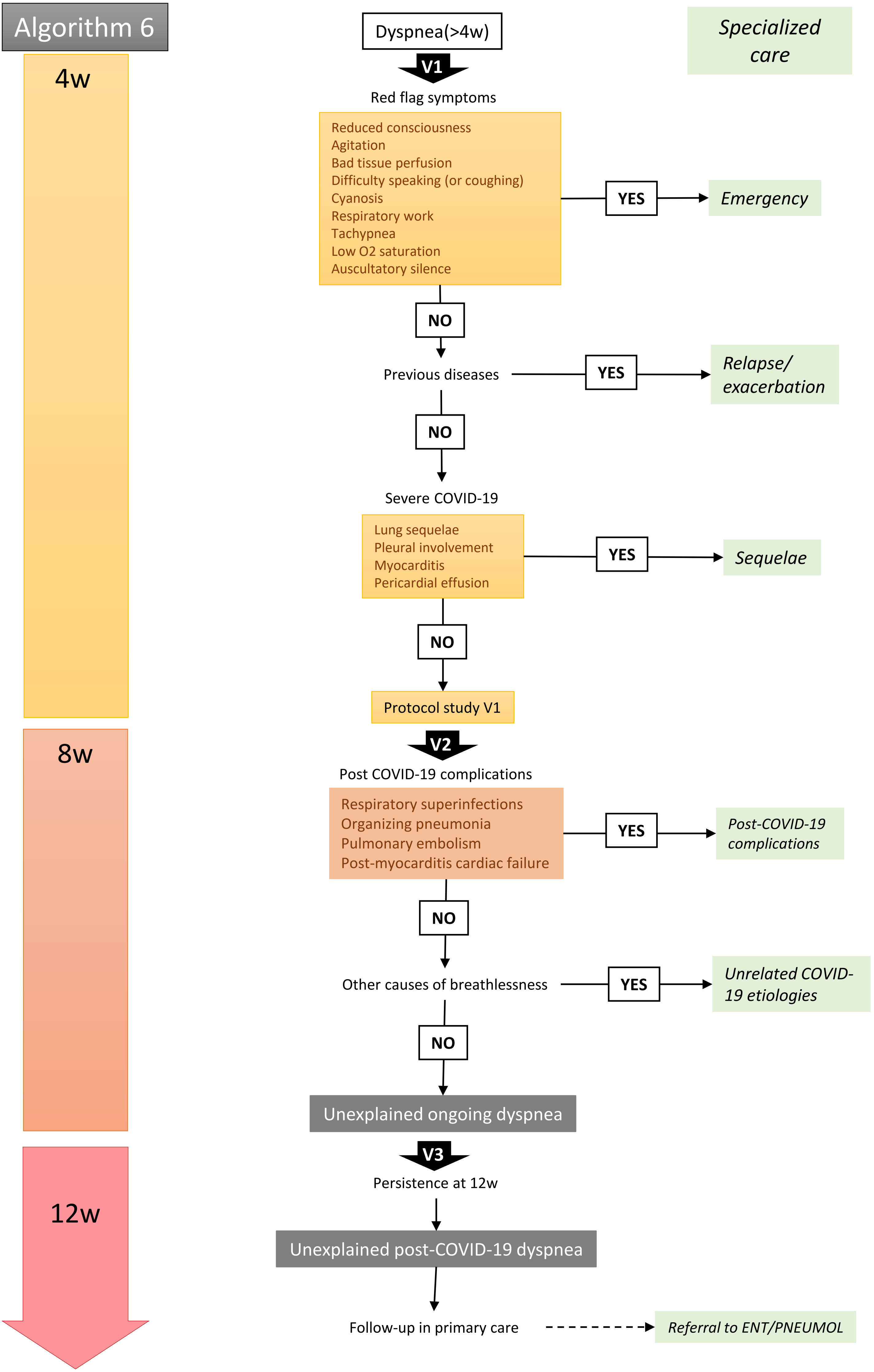

Algorithm 6
4w
8w
12w
Specialized care
Dyspnea(>4w)
V1
Red flag symptoms
Reduced consciousness
Agitation
Bad tissue perfusion
Difficulty speaking (or coughing)
Cyanosis
Respiratory work
Tachypnea
Low O2 saturation
Auscultatory silence
YES
Emergency
NO
Previous diseases
YES
Relapse/ exacerbation
NO
Severe COVID-19
Lung sequelae
Pleural involvement
Myocarditis
Pericardial effusion
YES
Sequelae
NO
Protocol study V1
V2
Post COVID-19 complications
Respiratory superinfections
Organizing pneumonia
Pulmonary embolism
Post-myocarditis cardiac failure
YES
Post-COVID-19 complications
NO
Other causes of breathlessness
YES
Unrelated COVID-19 etiologies
NO
Unexplained ongoing dyspnea
V3
Persistence at 12w
Unexplained post-COVID-19 dyspnea
Follow-up in primary care
Referral to ENT/PNEUMOL

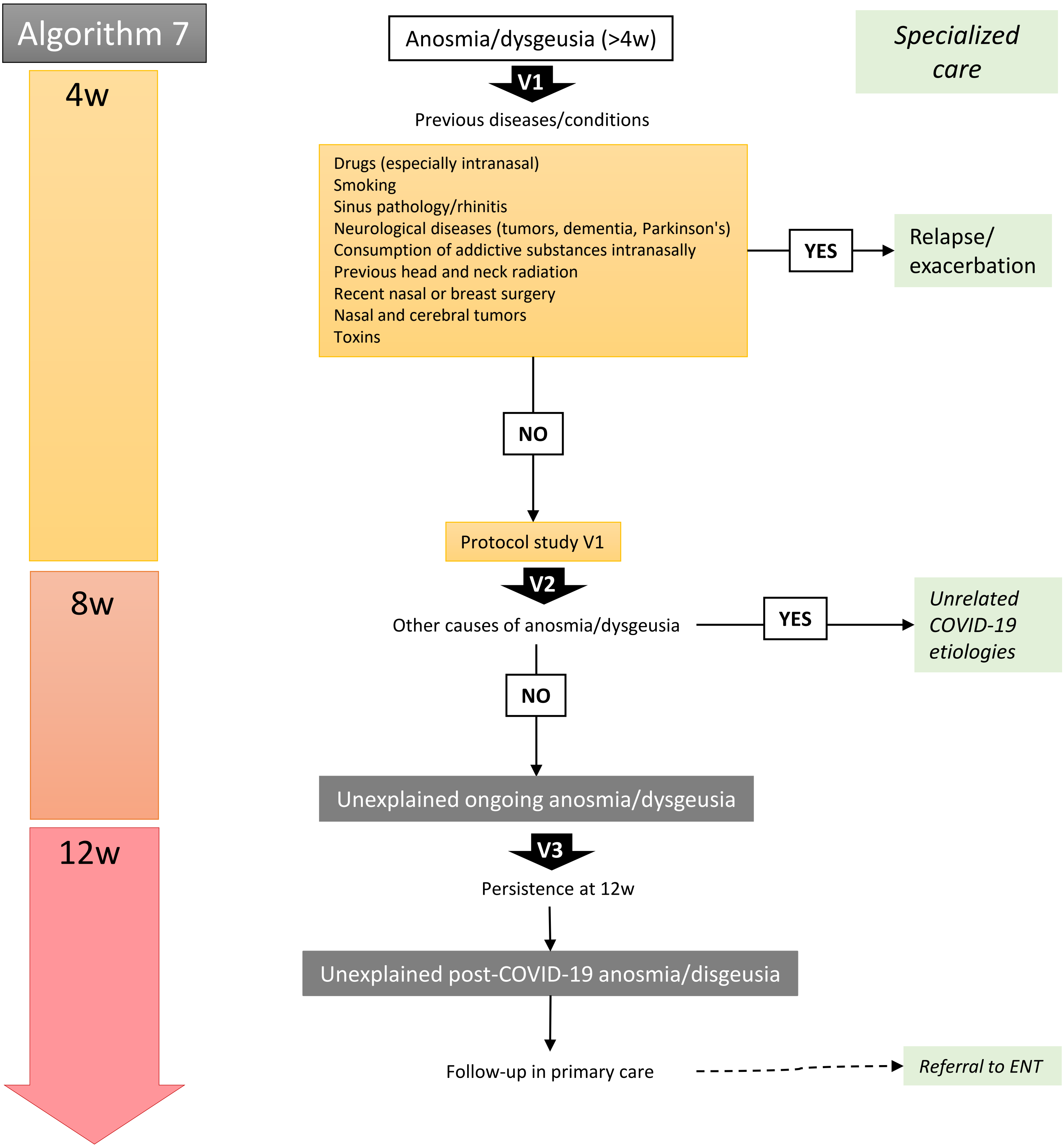
Algorithm 7
4w
8w
12w
Anosmia/dysgeusia (>4w)
V1
Previous diseases/conditions
Specialized care
Drugs (especially intranasal)
Smoking
Sinus pathology/rhinitis
Neurological diseases (tumors, dementia, Parkinson's)
Consumption of addictive substances intranasally
Previous head and neck radiation
Recent nasal or breast surgery
Nasal and cerebral tumors
Toxins
YES
Relapse/ exacerbation
NO
Protocol study V1
V2
Other causes of anosmia/dysgeusia
YES
Unrelated COVID-19 etiologies
NO
Unexplained ongoing anosmia/dysgeusia
V3
Persistence at 12w
Unexplained post-COVID-19 anosmia/disgeusia
Follow-up in primary care
Referral to ENT

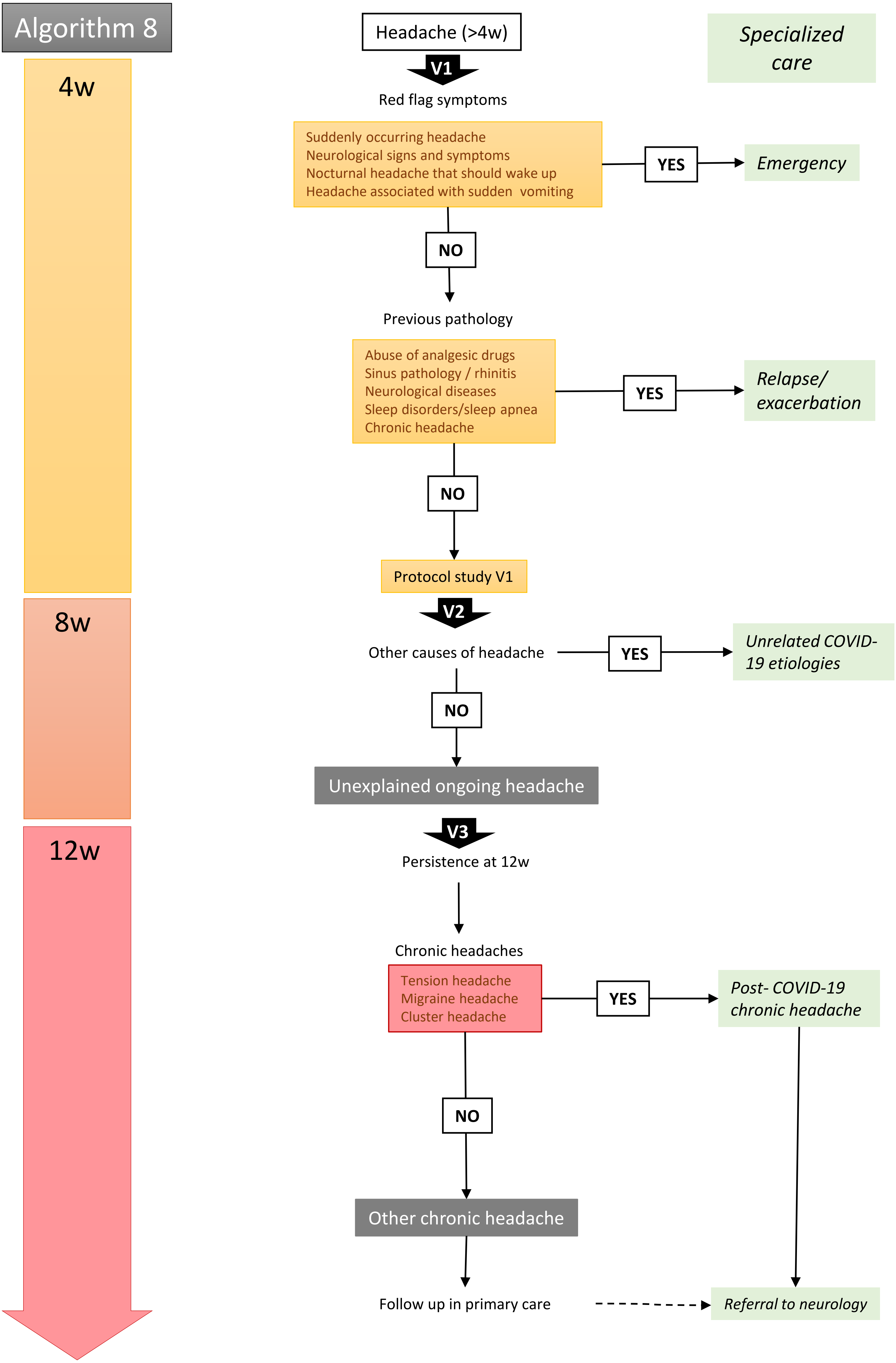

Algorithm 8
4w
8w
12w
Headache (>4w)
V1
Red flag symptoms
Suddenly occurring headache
Neurological signs and symptoms
Nocturnal headache that should wake up
Headache associated with sudden vomiting
YES
Emergency
Specialized care
NO
Previous pathology
Abuse of analgesic drugs
Sinus pathology / rhinitis
Neurological diseases
Sleep disorders/sleep apnea
Chronic headache
YES
Relapse/ exacerbation
NO
Protocol study V1
V2
Other causes of headache
YES
Unrelated COVID-19 etiologies
NO
Unexplained ongoing headache
V3
Persistence at 12w
Chronic headaches
Tension headache
Migraine headache
Cluster headache
YES
Post- COVID-19 chronic headache
NO
Other chronic headache
Follow up in primary care
Referral to neurology

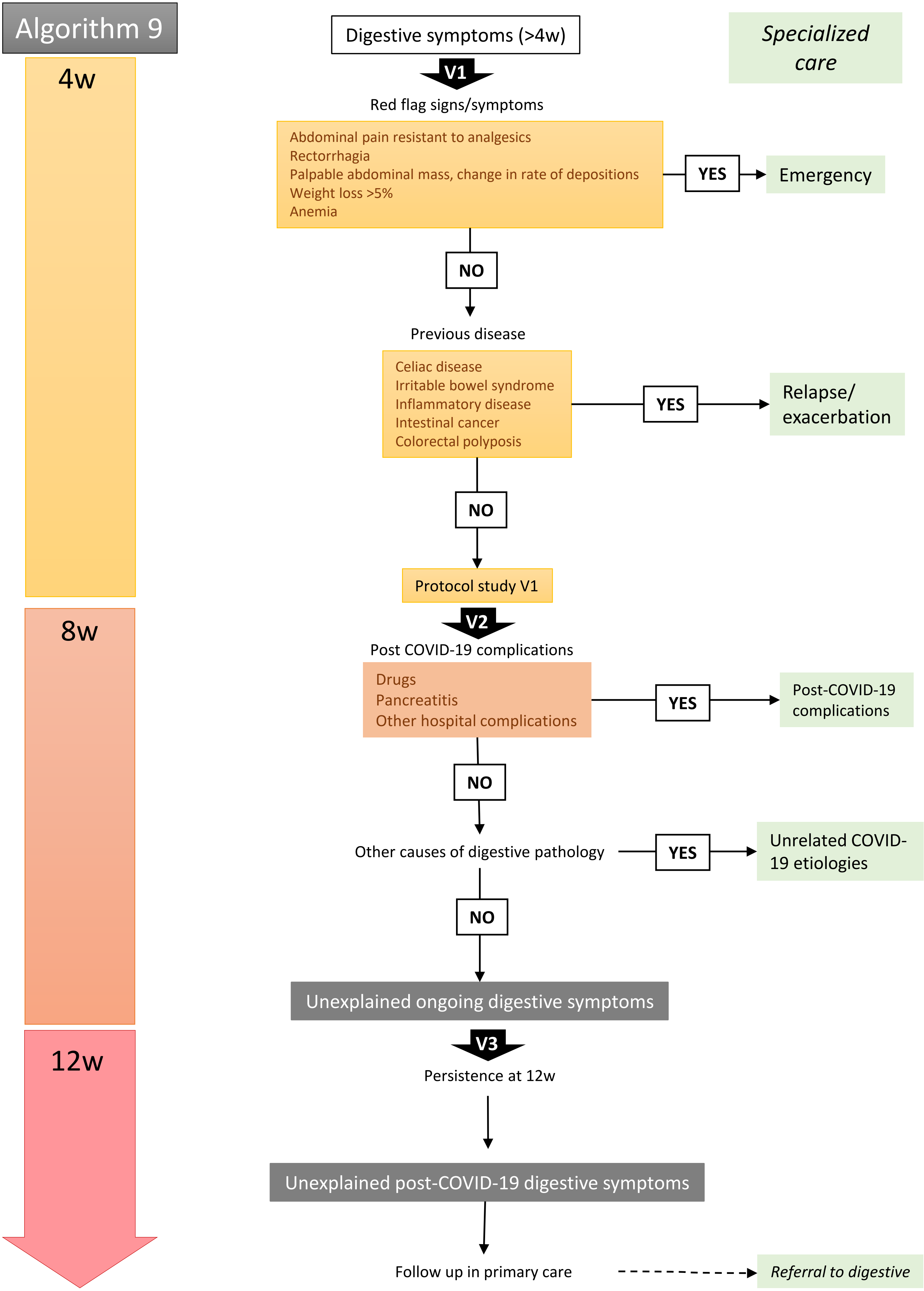

Algorithm 9
4w
8w
12w
Digestive symptoms (>4w)
V1
Red flag signs/symptoms
Abdominal pain resistant to analgesics
Rectorrhagia
Palpable abdominal mass, change in rate of depositions
Weight loss >5%
Anemia
YES
Emergency
NO
Previous disease
Celiac disease
Irritable bowel syndrome
Inflammatory disease
Intestinal cancer
Colorectal polyposis
YES
Relapse/ exacerbation
NO
Protocol study V1
V2
Post COVID-19 complications
Drugs
Pancreatitis
Other hospital complications
YES
Post-COVID-19 complications
NO
Other causes of digestive pathology
YES
Unrelated COVID-19 etiologies
NO
Unexplained ongoing digestive symptoms
V3
Persistence at 12w
Unexplained post-COVID-19 digestive symptoms
Follow up in primary care
Referral to digestive
Specialized care
